# Excessive gestational weight gain and emotional eating are positively associated with postpartum depressive symptoms among taiwanese women

**DOI:** 10.1186/s12905-023-02625-4

**Published:** 2023-09-01

**Authors:** Chia-Hsun Wu, Meei-Ling Gau, Su-Fen Cheng, Tzu-Ling Chen, Chih-Jung Wu

**Affiliations:** 1 Obstetrician and gynecologist, Department of Obstetrics and Gynecology, Cheng-Hsin General Hospital, Taipei, Taiwan; 2 Department of Nurse-Midwifery and Women’s Health, National Taipei University of Nursing and Health Sciences, 365, Ming-Te Rd, Peitou District, 11219 Taipei, Taiwan; 3 Department of Allied Health Education and Digital Learning, National Taipei University of Nursing and Health Sciences, Taipei, Taiwan; 4 School of Nursing, China Medical University, Taichung, Taiwan

**Keywords:** Gestational weight gain, Postpartum women, Emotional eating, Eating behavior, Postpartum depressive symptoms

## Abstract

**Background:**

Excessive gestational weight gain and emotional eating may be associated with postpartum depression symptoms. This study was designed to identify how gestational weight gain and eating behaviors are related to postpartum depression (PPD) symptoms among women in Taiwan.

**Methods:**

A cross-sectional study was conducted from March 2022 to October 2022 with 318 postpartum women recruited in Taipei, Taiwan. Gestational weight gain (GWG) for the total pregnancy period was recorded as inadequate, adequate, or excessive, based on the 2009 Institute of Medicine recommendations (IOM), accounting for pre-pregnancy body mass index category. Eating behavior at one month postpartum was measured on a 16-item 5-point Likert scale with three subscales: uncontrolled, restrained, and emotional. Maternal depressive symptoms were measured using the Edinburgh Postnatal Depression Scale with a cutoff score of 13.

**Results:**

The prevalence of postpartum depression symptoms (Edinburgh Postnatal Depression Scale ≥ 13) was 23.9% at one month postpartum. Logistic regression analysis revealed that excessive gestational weight gain and emotional eating were positively associated with postpartum depression symptoms at that time.

**Conclusion:**

Evidence presented here suggests that emotional eating and excessive GWG are associated with PPD symptoms in a Taiwanese population. In addition, it should be a public health priority to ensure a particular focus on mental health during the postpartum period. Healthcare providers should discourage pregnant women from unhealthy eating habits by targeting appropriate GWG and focusing on demand eating to reduce PPD in the postpartum period.

## Introduction

Postpartum depression (PPD) is an important public health issue that impacts postpartum women, affects parent-child interactions, and disrupts the harmony of family relationships. According to published statistics, PPD peaks 1 month after childbirth, with an incidence rate of 8.2–38.2% [1]. Fluctuations in body weight during the course of pregnancy and childbirth may affect the mental health of postpartum women [2]. Furthermore, recent empirical studies suggest that unhealthy weight gain during pregnancy coupled with poor birth outcomes and changes in body shape may have negative effects on self-esteem and body image, leading to psychological depression [3].

In Western countries, weight gain in pregnant women is commonly classified based on the 2009 Institute of Medicine (IOM) guidelines [4]. Both excessive and insufficient weight gain during pregnancy have been linked to a higher incidence of PPD than is associated with healthy weight gain [5, 6]. These relationships may involve impacts on levels of cortisol and insulin and on the hypothalamic-pituitary-adrenal axis, which can precipitate depression [7, 8]. Despite near-universal uptake of prenatal exams (98.2% of expecting mothers had ≥ 4 and 93.8% had ≥ 8 tests) in Taiwan in 2019 [9], associations between IOM-classified gestational weight changes and PPD are poorly understood.

Postpartum women tend to increase their intake of high-calorie and fatty foods as they adapt to multiple roles, and this affects the balance of their body’s satiety center [10]. They resort to emotional overeating and comfort eating [11, 12], consuming more food than their bodies require [13]. However, tools to assess early postpartum eating in Asian women are limited. Consuming more fruit, vegetables and dietary supplements is associated with lower depression at 2 months postpartum. In contrast, higher intake of high-sugar and fast food (such as cookies, French fries, and sugary drinks) has been linked to worsened depression symptoms [14, 15]. A previous study by Yu et al. (2022). found that emotional overeating and other unhealthy eating behaviors were positively associated with postpartum depression (PPD) in women 6–18 months after childbirth [16]. Research has also indicated concerning links between early postpartum eating habits, such as emotion-driven eating and problematic eating attitudes requiring psychiatric attention, and PPD [2]. These findings highlight a potential connection between postpartum women’s eating patterns and their mental health.

The elucidation of relationships between gestational weight gain, eating behaviors, and depression in postpartum women, especially in the early postpartum period and among Asian populations, may help decrease postpartum depression incidence and improve women’s postpartum health outcomes. The objectives of this study were to investigate the connections between excessive gestational weight gain (GWG) and emotional eating with PPD symptoms among postpartum women.

## Methods

### Study design, study settings and study participants

A cross-sectional study design with convenience sampling was used, in which study participants were recruited at two hospitals and four clinics in Taipei, Taiwan at 3–5 days postpartum. Data were collected from March 2022 through October 2022.Eligible participants were postpartum women aged ≥ 20 years, primipara, singleton, who had a healthy term infant (> 37 weeks), were living with their spouse, and were able to complete online structured questionnaires in Mandarin Chinese at one month postpartum. Women with gestational diabetes, pregnancy-induced hypertension, who were preterm < 37 week or had a clinical depression diagnosis were excluded.

Of the 345 recruited women, 27 did not complete the questionnaire and were dropped from the study, thus a total of 318 postpartum women were enrolled. Since the recruitment of postpartum mothers only during the period 3–5 days after delivery may have introduced selection bias, we attempted to minimize this possibility by establishing strict inclusion and exclusion criteria. To accommodate between-participant variation across various days postpartum, we also included days after delivery as a covariate in the analyses.

This study was approved by the institutional review board at the National Yang-Ming University (YM110204E).

### Sample size estimation

Based on the study of Polit and Beck [17], the suggested average effect size is 0.3 in an observational study. We used G*Power software (3.1.7) for Windows to estimate the required sample size for our study based on a two-sided α of 0.05, an effect size of 0.3 and a power of 0.8. Estimated required sample size under these parameters was 180, and our realized sample size of 318 was thus suitable for the present analysis.

### Measurements and data collection

Analyzed participant characteristics included demographic information and pre-pregnancy body mass index (BMI). Taiwanese health policy follows the 2009 Institute of Medicine (IOM) gestational weight gain guidelines [4] as established by the American College of Obstetricians and Gynecologists (ACOG, 2009), which are primarily based on pre-pregnancy BMI values calculated at the first prenatal visit [18]. We used self-reported maternal body weight and height to calculate the participants’ pre-pregnancy BMI, which was categorized as follows: underweight (< 18.5 kg/m^2^), normal weight (18.5–24.9 kg/m^2^), overweight (25.0–29.9 kg/m^2^), and obese (≥ 30 kg/m^2^).

In accordance with the 2021 Taiwanese national guidelines, maternal weight was assessed 14 times during pregnancy between the first antenatal visit (6–8 weeks) and 1 week before delivery [19]. GWG for the total pregnancy period was measured by doctors and nurses in the prenatal clinics and obtained from the maternal health booklet at the term of pregnancy. Subsequently, our GWG was compared with 2009 IOM guidelines for pre-pregnancy BMI: underweight (12–18 kg GWG), normal weight (11.5–16 kg GWG), overweight (7–11.5 kg GWG) and obese (5–9 kg GWG) women. GWG was recorded as inadequate if it was below the 2009 IOM recommendations, adequate if within the recommended range, and excessive if above the recommendation, accounting for the contributing role of each pre-pregnancy BMI category.

Eating behaviors were measured using a scale developed for this study, which had three subscales: uncontrolled, restrained, and emotional. This was a self-report scale (the items are listed in Table 1). Uncontrolled eating was defined as a tendency to overeat, with the feeling of being out of control and more intake than the internal demand. For examples: we modified item 1 to “I always felt hungry and wanted to eat.” Restrained eating was defined as a tendency not to overeat, with a feeling of being in control because of the conscious restriction of food intake to control body weight or to promote weight loss. Thus, we designed Item 8: “I deliberately ate small portions of food to control my weight.” Emotional eating was defined as the tendency to eat in response to negative emotions. In our study, we used Item 13: “I would eat more when I felt anxious.” The scale included 16 items rated on a 5-point Likert-type scale (0–4) for how frequent a certain behavior was, with a possible total score range of 0–64. Items relating to uncontrolled and emotional eating were coded as follows: 0: never, 1: seldom, 2: sometimes, 3: frequently, and 4: always (Table 1). Higher scores indicated that participants were eating more at 1 month postpartum.

**Table 1:** Exploratory factor analysis of the eating behavior scale at one month postpartum

	Mean (SD)	Factor Loading		
Percentage of variance explained		Uncontrolled	Restrained	Emotional
Variable		33.7%	19.9%	12.0%
1. I always felt hungry and wanted to eat.	1.70 (1.1)	**0.87**	0.01	0.15
2. I often felt hungry.	1.77 (1.2)	**0.87**	-0.01	0.07
3. I felt like my stomach was a bottomless pit (I kept wanting to eat).	2.00 (1.1)	**0.85**	0.04	0.15
4. I never felt full without emptying my plate.	1.42 (1.2)	**0.83**	0.02	0.16
5. I could eat again even after I had just eaten and felt full.	1.44 (1.1)	**0.78**	0.01	0.13
6. I wanted to eat whenever I saw someone else eating.	1.36 (1.1)	**0.74**	-0.02	0.18
7. I felt that eating was a pleasure.	1.97 (1.2)	**0.52**	0.01	0.31
8. I deliberately ate small portions of food to control my weight.	2.49 (1.1)	0.03	**0.89**	-0.08
9. I deliberately avoided eating the types of food that would make me gain weight.	2.35 (1.1)	0.06	**0.88**	-0.02
10. To control my weight, I would not eat even if I was hungry.	2.72 (1.0)	-0.02	**0.82**	-0.09
11. I was afraid to eat for fear of gaining weight.	2.68 (1.2)	-0.10	**0.69**	-0.05
12. I tried not to eat food with a high salt, sugar, and flavoring content.	2.32 (1.2)	0.03	**0.59**	-0.01
13. I would eat more when I felt anxious.	1.16 (1.0)	0.19	-0.05	**0.90**
14. I would eat more when I felt lonely.	1.03 (1.0)	0.24	-0.01	**0.88**
15. I would eat more when I was in a bad mood.	1.35 (1.1)	0.20	-0.13	**0.82**
16. I deliberately ate as much as possible when I felt unhappy.	0.74 (0.8)	0.12	-0.10	**0.63**

The content validity of eating questionnaire was assessed by six experts: a psychiatrist, an obstetrician, two obstetrical experts, and two nutritionists. These specialists rated the correctness, appropriateness, and clarity of the questionnaire using a 4-point Likert-type scale ranging from 1 (very poor) to 4 (very good). The content validity index of the eating questionnaire was determined by dividing the number of specialists giving an item a rating of 3 or 4 by the total number of specialists and then averaging the item-level results, which yielded a result of 0.95.

We conducted exploratory factor analysis using principal components following varimax rotation with Kaiser normalization on the eating questionnaire responses. Initial factors were identified as factors with eigenvalues > 1 based on examination of the scree plot. The percentage of variance in the eating questionnaire responses explained by the initial factors was 65.7% (Table 1). The results of the factor analysis supported the construct validity of the scale. The internal reliability (Cronbach’s α) of the eating questionnaire was acceptable (0.82 for the whole scale, 0.90 for uncontrolled, 0.83 for restrained, and 0.86 for emotional eating).

### Postpartum depression

We assessed prenatal depression using the 10-item Chinese version of the Edinburgh Postnatal Depression Scale (EPDS) with a cut-off score of 13 for depression symptoms [20]. This scale is the most widely used self-reporting screening tool, based on a 4-point Likert-type scale (total score range: 0–30). Higher scores indicate more depressive symptomatology [21]. A cutoff score of ≥ 13, indicating the likelihood of major depressive symptoms, has been suggested for Taiwanese women [20, 22]. Cronbach’s α was 0.87 at 1 month postpartum in this study.

### Data analysis

We analyzed the data using Statistical Package for the Social Sciences for Windows version 22.0 (SPSS, Chicago, IL, USA). We used χ^2^ and independent samples *t* tests to identify and examine differences in the characteristics of the participants between the with PPD and without PPD groups. Ambler and Royston [23] suggested that using a *p* value of less than 0.10 to screen for independent variables when the sample size was less than 500 may avoid missing significant predictive variables during the analyses. Therefore, we used *p* < 0.05 to identify variables related to PPD groups in the bivariate analyses. Binary logistic regression was used to identify factors and to examine the adjusted odds ratios (ORs) for PPD.

## Results

### Representativeness of study samples

Bilir et al. (2019) concluded that education level is a key socio-demographic factor affecting gestational weight gain [24]. The representativeness of our study sample in terms of maternal education level was checked by comparing the present sample to the national data on Taiwanese newborn infants whose mothers were at least 20 years of age. The results showed that our samples were not significantly different from the national dataset in terms of distribution of maternal education level (high school or below, university or higher; χ2 = 4.01, df = 1, p = 0.05) [25].

### Characteristics of the study population

The prevalence of PPD symptoms (EPDS ≥ 13) was 23.9% at 1 month postpartum. Mean maternal age of the 318 women was 36.3 ± 5.2 (range, 20–45 years). Most participants reported an educational level of university or higher (87.7%). We merged pre-pregnancy BMIs for the overweight and obesity categories into one group (overweight and obesity). Prevalence of underweight, normal, and overweight and obesity among women were 13.9%, 70.7%, and 15.4%, respectively. Mean GWG by postpartum women was 11.18 ± 4.8 kg. Most participants (142; 44.7%) had inadequate GWG (Table 2).

**Table 2:** Characteristics of the study participants (*n* = 318)

	Overall sample	EPDS < 12 (*n* = 242) *n* (%) Mean (SD)	EPDS ≥ 13 (*n* = 76) *n* (%) Mean (SD)	χ^2^/*t* test	*p* value
**Maternal age**				2.01	0.19
20–34	148	118 (48.8)	30 (39.5)		
≥ 35	170	124 (51.2)	46 (60.5)		
**Education level**				2.25	0.39
High school or below	34	23 (9.6)	11 (14.5)		
University or higher	283	218 (90.4)	65 (85.5)		
**Pre-pregnancy BMI (kg/m** ^**2**^ **)**				1.44	0.70
Underweight	44	31 (12.8)	13 (17.1)		
Normal	225	174 (71.9)	51 (67.1)		
Overweight and obesity	49	37 (15.3)	12 (15.8)		
**Delivery method**				0.01	0.93
Vaginal birth	223	170 (70.2)	53 (69.7)		
Cesarean birth	95	72 (29.8)	23 (30.3)		
**Weight gains according to IOM**				15.37	< 0.001**
Adequate	137	107 (44.2)	30 (39.5)		
Inadequate	142	115 (47.5)	27 (35.5)		
Excessive	39	20 (8.3)	19 (25.0)		
**Eating behaviors**					
Uncontrolled		11.02 (6.4)	13.67 (5.8)	0.68	0.002*
Restrained		12.72 (4.4)	12.04 (4.1)	0.77	0.23
Emotional		3.66 (3.1)	6.28 (3.4)	1.10	< 0.001**

* *p* < 0.05; ** *p* < 0.001

^a^ Fisher’s test; the expected frequencies are less than five in the table

### Eating behavior

Mean GWG in the uncontrolled, restrained, and emotional eating groups was 11.66 ± 6.4 (range 0–28), 12.56 ± 4.3 (range 0–20), and 4.28 ± 3.4 (range 0–13), respectively. Uncontrolled eating was reported by some of our study participants, and the item with the highest score was “I felt like my stomach was a bottomless pit (I kept wanting to eat).” The lowest score for the uncontrolled eating group was “I wanted to eat whenever I saw someone else eating” (Table 1). A medium level of restrained eating (mean score of > 2.5) was reported by some participants. Comments included “To control my weight, I would not eat even if I was hungry” and “I was afraid to eat for fear of gaining weight.” Emotional eating was reported by some participants, and the item with the highest score was “I would eat more when I was in a bad mood.” The lowest score for emotional eating was “I deliberately ate as much as possible when I felt unhappy” (see Table 1).

### Relationship between GWG, eating behavior and PPD

Women with and without PPD at 1 month postpartum differed significantly in GWG and in uncontrolled and emotional eating behavior. Women with excessive GWG reported a higher rate of PPD symptoms than those without excessive GWG (25% vs. 8.3%, *p* < 0.001). Women with PPD tended to have significantly higher mean uncontrolled eating scores (13.67 ± 5.8 vs. 11.02 ± 6.4, *p* = 0.002) and emotional eating scores (6.28 ± 3.4 vs. 3.66 ± 3.1, *p* < 0.001) than those without PPD at 1 month postpartum among our women (see Table 2). Other characteristics did not differ significantly between PPD symptoms and non-PPD symptoms.

We used a logistic regression model to identify factors associated with PPD at 1 month postpartum, see Table 3. All variables detailed in the Methods were included. The final multiple logistic regression results showed that model yielded a Cox and Snell *r*^2^ = 0.14, Nagelkerke *r*^2^ = 0.20, and a Hosmer-Lemeshow goodness-of-fit χ^2^ = 12.6, *df* = 8, *p* = 0.13. Women with excessive GWG were more likely to reach PPD at 1 month postpartum (OR: 2.47; 95% CI: 1.05–5.81), and emotional eating was positively associated with PPD at 1 month postpartum (OR: 1.23; 95% CI: 1.12–1.35).

**Table 3:** Logistic regression model of postpartum depressive symptoms (EPDS ≥ 13) at 1 month (*n* = 318)

Variable	Crude OR	95% CI	*p* value	Adjusted OR	95% CI	*p* value
**Maternal age**						
20–34	1			1		
≥ 35	1.46	0.86–2.47	0.16	1.67	0.92–3.02	0.09
**Pre-pregnancy BMI (kg/m** ^**2**^ **)**						
Underweight	1			1		
Normal	0.70	0.34–1.43	0.33	0.54	0.25–1.20	0.13
Overweight and obesity	0.77	0.31–1.94	0.58	0.64	0.23–1.77	0.39
**GWG**						
Adequate	1			1		
Inadequate	0.84	0.49–1.50	0.55	0.83	0.45–1.55	0.56
Excessive	3.39	1.61–7.15	0.001*	2.47	1.05–5.81	0.04*
**Eating behaviors**						
Uncontrolled	1.03	0.98–1.08	0.32	1.02	0.97–1.07	0.55
Restrained	0.98	0.92–1.04	0.45	0.96	0.90–1.03	0.26
Emotional	1.24	1.13–1.36	< 0.001**	1.23	1.12–1.35	< 0.001**

* *p* < 0.05; ** *p* < 0.001

The adjusted model was adjusted for demographic variables and pre-pregnancy BMI.

## Discussion

The reported incidence of PPD varies greatly between studies, with values of 6.8–30% reported for various countries around the world [1]. In the present study, we found that the incidence of PPD 1 month after childbirth was 23.9%, which is higher than the previously reported rates of 13.8–17.3% for Chinese women and 19% for Taiwanese women [1]. This high incidence of PPD indicates a need to implement routine assessment and management of women’s postpartum mental health. We used a cutoff value of EPDS ≥ 13 to classify PPD, differing from previous studies in which cutoffs of 10 and 13 were used to define minor and major depressive symptoms, respectively [21, 26]. Because we excluded participants with clinically diagnosed depression in this study, our assessment of depression and its prevalence among the study participants does not represent a diagnosis and should be regarded only as a general estimate. Accordingly, our findings cannot be generalized to women who have depression, and we recommend that population-based epidemiological studies be conducted to further explore psychological depression in postpartum women.

We found that excessive GWG is a significant predictor of PPD within the first month following childbirth, which aligns with earlier studies indicating that excessive GWG can lead to pregnancy complications, adverse birth outcomes, a change in self-identity, and depression [3]. Based on a recent empirical study, which used the 2009 IOM guidelines that has been adopted worldwide [4], 27.8% of pregnant women gained too much weight and 39.4% gained insufficient weight. A global survey of pregnant women revealed that women in North America and Asia respectively exhibited the greatest (14.74 kg) and smallest (11.36 kg) extents of weight gain [27]. Earlier research revealed that excessive weight gain during pregnancy affects women’s body confidence. Medical professionals in Taiwan recommend a pregnancy weight gain of 10–14 kg [18, 28]. It is hypothesized that women who experience excessive weight gain during pregnancy may face additional stress related to managing their weight, resulting in increased dissatisfaction with their body shape [2]. In Taiwan, which has a high level of economic development, women tend to have children later in life than in less economically developed societies [25]. On the one hand, this may lead them to tend to reduce their physical activity and increase their food intake in the hope of ensuring favorable birth outcomes [29]. They may thus unintentionally gain excessive weight during their pregnancy, even when healthcare professionals are helping them to control their GWG [30]. On the other hand, 44.7% of Taiwanese women exhibited insufficient weight gain during pregnancy in the previous study [28]. In the interview period, we found that the mass media promoted the concept of nourishing the fetus without gaining fat, and normalized the notion of gaining less weight than the recommended amount during pregnancy. Recent medical research using the GWG standards established by the IOM has revealed that women who experience insufficient GWG during pregnancy have a significantly higher risk of poor fetal growth and development and preterm birth. As GWG plays a key role in pregnancy, avoiding either excessive or insufficient GWG during this period should be emphasized in future prenatal health education.

Although the present cross-sectional study provides an incomplete picture of the association between pregnancy weight gain and PPD, our findings offer credible evidence for such an association, since the gestational weight data we used were obtained from records kept at medical clinics. In the future, more emphasis should be placed on the importance of appropriate weight gain during pregnancy to promote maternal health and to help to prevent PPD [2, 3], particularly among those who experience excessive weight gain during pregnancy.

Previous research has indicated that low mood is associated with reduced initiative to make lifestyle changes, a decline in food preparation standards, and a lack of engagement in healthy eating behaviors [14, 31, 32]. A prospective study by Baskin and Hill [14] suggested that unhealthy dietary intake during pregnancy is associated with psychological depression during pregnancy, but not at 3 months postpartum. Another recent physiological study revealed that elevated levels of the postpartum neurohormones dopamine, serotonin, and norepinephrine affect the appetite of pregnant women and increases the incidence of psychological depression [33], but it is unclear whether this effect also applies to the early postpartum period. Accordingly, the temporal continuity between eating behaviors and depression warrants further investigation.

We found that women exhibiting emotional eating behaviors had a significantly higher incidence of PPD. A systematic review in 2020 [15] reported that women with depression who scored above the EPDS cutoff of 13 points exhibited poorer single-nutrient supplementation behavior, were less likely to choose healthy foods [32], and tended to choose more foods that were high in sugar and fat [34]. However, the methods used to measure eating behavior in previous studies varied, and to our knowledge there are no studies examining the association between emotional eating behavior and PPD in women at 1 month after childbirth. Consequently, we are unable to compare our results with those of previous studies.

Nevertheless, Emerson et al. [35] found that US women who were diagnosed with PPD at 0–13 months after giving birth had higher scores for emotional eating, which was measured via questions such as: “Do you have the desire to eat when you are irritated?”, “Do you have a desire to eat when you are feeling lonely?”, and “Do you get the desire to eat when you are anxious, worried or tense?” Because the measurements that were used to assess emotional eating were similar to those of the present study but yielded different results, the direction of the association between emotional eating and PPD deserves further investigation.

In the present study, the score for uncontrolled eating behavior was significantly higher for participants with PPD, but the regression model did not show a significant association, possibly because of the relatively strong correlation between emotional eating and PPD. European studies have shown that people with depression tend to exhibit irrational eating behavior and poor ability to regulate their portion sizes and mealtimes, leading to increased uncontrolled eating [31]. Among the questions assessing uncontrolled eating, the items “I felt like my stomach was a bottomless pit (I kept wanting to eat),” and “I felt that eating was a pleasure” scored the highest. This result is consistent with the empirical survey findings of Bijlholt et al. [36] and Paans et al. [31], which showed that uncontrolled eating is an unhealthy behavior that disrupts the physiological need for self-satiety, excessively increases the appetite, and carries the risk of future obesity [31, 36]. Therefore, when designing future dietary education interventions for postpartum women, emphasis should be placed on encouraging them to focus on their physiological nutritional needs, in order to prevent excessive and compulsive eating.

Previous studies have suggested that women who are overweight or obese prior to pregnancy tend to be concerned about postpartum weight retention, leading to an increase in restrained eating behaviors [36]. A British study on individuals with eating disorders (e.g., eating restraint, eating concern, shape concern, and weight concern) reported a higher incidence of depression [37]. Women are conscious of changes in body weight and often resort to restricting their diet and eating less to maintain body shape and weight; this shares similarities with the concept of restrained eating described by the authors. However, in the present study, we did not find an association between higher levels of restrained eating and PPD, possibly because most of the participants had normal BMIs before pregnancy.

An American study found that women’s eating behavior did not change significantly during the first 6–18 months following childbirth [16], although some researchers have speculated that postpartum eating behavior may impact mental health [14]. In the present study, we extended the scope to investigate the relationship between pre-existing eating patterns, food intake behavior, and psychological depression [14, 32, 36]. However, since we used a cross-sectional design, we were unable to determine the direction of causality (i.e., whether increased disordered eating behaviors lead to depression, or whether depression affects eating behavior). Regardless, our study provides evidence that non-emotional eating behaviors should be encouraged in the postpartum phase. More research is needed to examine the association between eating behavior and mental health further among women in different postpartum phases, particularly during the early phase. Health professionals should gain a deeper understanding of women’s eating behaviors and assess their mental well-being during postpartum medical checkups and home visits to ensure their optimal physical and mental health.

The dietary questionnaire we used was developed specifically for this study, and demonstrated acceptable construct validity and internal consistency for assessing three aspects of eating behavior (uncontrolled, restrained, and emotional eating). Previous studies have supported the importance of these three dimensions in assessing the eating behavior of women [31, 36]. While we placed special emphasis on personal food obsessions, adopted behavior, and eating behavior, earlier studies have shown that family and eating environments are also important determinants of eating behavior [38]. We recommend that future studies incorporate additional aspects such as these into the measurement of eating behavior. Conducting longitudinal surveys of eating behavior at several time points from pregnancy to postpartum could also help to provide a deeper understanding of the correlation between eating behavior and psychological depression.

This study was subject to some limitations. First, the metric of GWG over the total pregnancy period was measured in the clinic by doctors and nurses and obtained from the maternal health booklet. We did not verify the reliability and validity of the anthropometric measures, but assumed that these were acceptable. In addition, the 2009 IOM GWG guidelines for pre-pregnancy BMI were developed using global data, and have not been validated for the more specific case of Taiwanese women. Accordingly, there exists the possibility of misclassification of the pre-pregnancy BMI in an Asian population [39]. Second, our study participants appear to be older and have higher educational levels than samples in previous studies. However, the distribution of those characteristics is not very different from other studies conducted in Taipei, the capital of Taiwan. The prevalence of 15.4% for overweight/obese pre-pregnant women and 23.9% for PPD symptoms at 1 month postpartum in the study are similar to those in previous studies [1, 40, 41], suggesting the generalizability of the findings. Given the measurements and data analyses performed, the associations reported in the current study should be considered correlational, and future longitudinal studies with a longer follow-up period are needed to establish causal relationships.

## Conclusions

Women with excessive GWG exhibited increased depressive symptoms, and postpartum women with higher emotional eating behaviors may be at high risk for postpartum depression. Healthcare providers should support pregnant women by promoting appropriate gestational weight gain. The incidence of a tendency to overeat in response to negative or depressive moods and of postpartum depression among postpartum women in Taiwan indicates that there is a need for the development of strategies to improve the health of this population.

## Data Availability

The data and materials that support the findings of this study are available from the corresponding author upon request.

## Declarations

### Abbreviations

BMI, body mass index.

EPDS, Edinburgh Postnatal Depression Scale.

GWG, gestational weight gain.

IOM, Institute of Medicine.

PPD, postpartum depression.
